# Prodigiosin Induces Autolysins in Actively Grown *Bacillus subtilis* Cells

**DOI:** 10.3389/fmicb.2016.00027

**Published:** 2016-01-28

**Authors:** Tjaša Danevčič, Maja Borić Vezjak, Maja Tabor, Maša Zorec, David Stopar

**Affiliations:** ^1^Chair of Microbiology, Department of Food Science and Technology, Biotechnical Faculty, University of LjubljanaLjubljana, Slovenia; ^2^Chair of Microbiology and Microbial Biotechnology, Department of Animal Science, Biotechnical Faculty, University of LjubljanaLjubljana, Slovenia

**Keywords:** prodigiosin, autolysis, *Bacillus subtilis*, antimicrobial, mechanism, autolysin, lytic rate, comet assay

## Abstract

Prodigiosin produced by marine bacterium *Vibrio ruber* DSM 14379 exhibits a potent antimicrobial activity against a broad range of Gram positive and Gram negative bacteria. The mechanism of prodigiosin antimicrobial action, however, is not known. In this work, the effect of prodigiosin on *Bacillus subtilis* growth, cell membrane leakage, and induction of autolysins was studied. Treating *B. subtilis* with prodigiosin resulted in rapid decline of optical density and increased cell membrane leakage measured by β-galactosidase activity. Cell lysis was initiated immediately after treatment with prodigiosin in the middle exponential phase and was completed within 2 h. Lytic activity of prodigiosin in mutant strains with impaired autolysin genes *lytABCD* decreased for 80% compared to the wild type strain, while in *lytABCDEF* mutant strain prodigiosin had no bacteriolytic but only bacteriostatic effect. Fast prodigiosin lytic activity on individual *B. subtilis* cells was confirmed by a modified comet assay. The results indicate that prodigiosin autolysin induction in *B. subtilis* is growth phase dependent.

## Introduction

Prodigiosin, a secondary metabolite, is produced by several bacterial genera including *Serratia, Streptomyces, Vibrio, Hahella, Zooshikella*, and *Pseudoalteromonas* (Farmer et al., 1988; Sawabe et al., 1998; Shieh et al., 2003; Williamson et al., 2006; Kumar and Nair, 2007; Fehér et al., 2010; Starič et al., 2010; Lee et al., 2011; Stankovic et al., 2012). Prodigiosin provides numerous ecophysiological benefits for the producing cell and has a good potential for biotechnological as well as medical applications (Burger and Bennett, 1985; Hood et al., 1992; Ryazantseva et al., 1995; Seganish and Davis, 2005; Haddix et al., 2008; Starič et al., 2010; Borić et al., 2011). Prodigiosin has been used in control of plant diseases caused by bacteria and fungi (Okamoto et al., 1998; Someya et al., 2001; Meschke et al., 2012). It has affinity to DNA (Melvin et al., 2000), but shows no *in vitro* or *in vivo* genotoxic effect on *Salmonella typhimurium* cells (Guryanov et al., 2013). It modulates H^+^/Cl^-^ symport activity (Konno et al., 1998). When used as a coloring agent, it retains its antibiotic activity (Alihosseini et al., 2008). Prodigiosin has anticancer, antimalarial and immunosuppressant properties (Pérez-Tomás et al., 2003; Williamson et al., 2007). It inhibits growth of a wide range of Gram positive bacteria including *Bacillus subtilis* and *Staphylococcus aureus*, as well as Gram negative *Escherichia coli, Salmonella enterica*, and *Erwinia carotovora* (reviewed in Stankovic et al., 2014). The mechanism of its antibacterial action, however, is not understood on the molecular level. In this work, we study the molecular targets of prodigiosin in *B. subtilis*.

Many antibacterial agents target cell membrane and cell wall (Falk et al., 2010; Lacriola et al., 2013). In *B. subtilis* induction of autolysins and subsequent cell wall degradation is a major mechanism of antimicrobial action of several different compounds (Falk et al., 2010; Lacriola et al., 2013). Autolysins are enzymes involved in hydrolysis and remodeling of the peptidoglycan in the bacterial cell wall (Smith et al., 2000). It is absolutely critical to maintain the regulation of potentially suicidal activity of autolysins. The regulation of autolysin activity is mainly due to the local pH in respiring cells. In respiring cells, protons are extruded across the cytoplasmic membrane and bind to cell wall constituents (Jolliffe et al., 1981; Kemper et al., 1993; Calamita and Doyle, 2002). The protonation of the cell wall constituents create a wall matrix of low pH. Proton motive force sustains this local low pH and, in turn, autolytic activity is inhibited because *N*-acetylmuramyl-L-alanine amidase cannot function below pH 5.5. There are 36 autolysins present in the *B. subtilis* genome (Smith et al., 2000). Among different autolysins in *B. subtilis*, two autolysins of vegetative growth named LytC and LytD are responsible for about 95% of the bacterial cell autolysis during active growth (reviewed in Smith et al., 2000). It has been shown previously that β-galactosidase release correlates with activation of pathways leading to autolysis (Falk et al., 2007). Apart from β-galactosidase release several other methods (e.g., optical density, cell count) can be used to determine bacterial cell lysis. The comet assay is a method primarily used for the detection of DNA damage and cell lysis in a single cell (Ostling and Johanson, 1984; Singh et al., 1988). Recently, comet assay was applied to detect bacterial lysis after bacteriophage treatment (Khairnar et al., 2014). Using comet assay to detect single cell lysis provides an advantage over enzyme activity or optical density measurements of cell lysis which probe a population response.

In this work, the mechanism of antibacterial activity of prodigiosin on *B. subtilis* was studied. The ability of prodigiosin to affect bacterial population structure was determined in simple co-culture experiments. The effect of prodigiosin on the bacterial growth, cell wall integrity, autolysis, and DNA damage was determined on the wild type strain and several autolysin mutants. This study is the first attempt to determine the mechanism of antibacterial activity of prodigiosin in *B. subtilis in vivo*. The results indicate that prodigiosin activates rapid autolysis in *B. subtilis*.

## Materials and Methods

### Bacterial Strains, Mutant Strain Construction and Growth Conditions

Bacterial strains used in this study were *B. amyloliquefaciens* FZB42, *B. licheniformis* ATCC9445A, *B. subtilis* NCIB3610 all obtained from BGSC, *B. subtilis* ATCC6051, *B. mycoides* DSM2048 obtained from DSMZ, *B. subtilis* PS-216 *wt* (Stefanic and Mandic-Mulec, 2009), *B. subtilis* PS-216 *amyE::*P_hyperspank_*-mKate2 cat* (Stefanic et al., 2015), *Escherichia coli* MG1655 and *Vibrio ruber* DSM14379 (Stopar et al., 2004; Borić et al., 2011). In addition mutant strains of *B. subtilis* PS-216 *ΔlytABC::neo, B. subtilis* PS-216 *ΔlytABC::neo ΔlytD::tet, B. subtilis* PS-216 *ΔlytABC::neo ΔlytD::tet ΔlytE::cam, B. subtilis* PS-216 *ΔlytABC::neo ΔlytD::tet ΔlytE::cam ΔlytF::spc* and *B. subtilis* PS-216 *srfA-lacZ neo* were prepared. The *lyt* mutants were constructed by transforming the *B. subtilis* PS-216 *wt* strain with the DNA isolated from *B. subtilis* L16611 and *B. subtilis* L16628 obtained from BGSC (Margot and Karamata, 1992). The *srfA-lacZ* construct was made by transforming *B. subtilis* PS-216 *wt* strain with the DNA isolated from *B. subtilis* KTB308 (Bacon Schneider et al., 2002). *Bacillus* strains were grown at 37°C (except *B. mycoides* at 28°C) and 200 rpm in LB medium or CM medium (Albano et al., 1987) or at 28°C and 200 rpm in PYE medium with 3% (w/V) NaCl (Danevčič et al., 2005).

### Prodigiosin Extraction

*Vibrio ruber* DSM14379 was cultured overnight in PYE medium with 3% (w/V) NaCl at 28°C and 200 rpm (Danevčič et al., 2005). Overnight culture was diluted 100 times in 400 mL of fresh M9 medium supplied with 5 g/L glucose (Starič et al., 2010) and incubated for 16 h at the same growth conditions. Cells were harvested with centrifugation at 10397 *g* for 10 min. Prodigiosin was extracted from cells with an equal volume of acetone at 28°C and 200 rpm for 2 h. After centrifugation for 15 min at 10397 *g* to remove cell debris, the solvent was evaporated. Dry biomass was resuspended in sterile 96% (V/V) ethanol and the pigment extract was filtered through 0.20 μm filter. Prodigiosin concentration in the extract was determined as described previously by Starič et al. (2010). The prodigiosin purity in the extract was confirmed by HPLC using Kinetex C18 column (250 mm × 4.6 mm, 5 μm, Phenomenex, USA) according to the modified method described previously (Nakashima et al., 2006). The separation was achieved using 0.1% trifluoroacetic acid (A) and methanol (B) mobile phases, and a gradient elution program at 1 mL^-1^ min with the following parameters: 0–5 min 100% A, 5–25 min 0–100% B (linear gradient), 25–30 min 100% B (isocratic), and 30–50 min 100% A (isocratic) to re-equilibrate the column, monitored by UV-VIS detection. The purity was determined by HPLC at different wavelengths in the range from 400 to 600 nm. The HPLC elugram at 535 nm is given in supplementary materials (Supplementary Figure S1). In the tested range the extract contained more than 98% prodigiosin of both isomers α and β.

### Minimal Inhibitory Concentration Determination

Minimal inhibitory concentration (MIC) values were determined in 96-well microtiter plate assay according to CLSI standards (Clinical and Laboratory Standards Institute, 2009). Briefly, bacterial strains were grown overnight in LB medium at 28°C or 37°C. Inoculum was prepared by diluting overnight cultures in 0.9% NaCl to 0.5 McFarland (∼10^7^ cells per mL). Wells of microtiter plates were filled with 0.1 mL of the appropriate concentration of the prodigiosin, ethanol or water and 0.01 mL of the inoculum to have the approximate number of cells in each inoculated well 10^5^. The dilutions of prodigiosin, ethanol and water were made in LB medium. Prodigiosin was diluted in the final concentration range from 0.6 mg L^-1^ to 367.1 mg L^-1^, while ethanol was diluted in the final concentration range from 0.02 to 12.03% (V/V). For positive controls LB medium was diluted with the same amounts of sterile distilled water as ethanol. Microtiter plates were incubated without shaking at 37°C for 20 h and the optical density at 650 nm (OD_650_) was measured spectrophotometrically at the beginning and the end of incubation. The lowest prodigiosin concentration in the well with no growth after 20 h of incubation, was taken as the MIC value.

### Co-culture Experiments

*Bacillus subtilis* PS-216 *wt* was grown either in co-culture with *V. ruber* DSM14379 or in pure culture. For co-culture experiments, strains were separately grown in PYE medium with 3% (w/V) NaCl (*V. ruber*) or in LB medium (*B. subtilis*) until the stationary phase. Co-cultures were inoculated into PYE medium with 3% (w/V) NaCl with *V. ruber* DSM14379: *B. subtilis* PS-216 in the ratio 1:2 (V:V), the overall inoculum being 1% (V/V). CFU was determined at the beginning and 20 h after incubation at 28°C and 200 rpm. CFU values were determined on PYE plates without NaCl (for *B. subtilis* PS-216 *wt*) and PYE plates with 3% (w/V) NaCl (for both strains). Due to the red pigmentation of *V. ruber* DSM14379 the colonies were clearly distinguished. Malthusian fitness was calculated based on CFU counts according to Ahn et al. (2006). Prodigiosin concentration in co-cultures was determined as described previously in Borić et al. (2011).

### Cell Treatment with Prodigiosin in Different Growth Phases

*Bacillus subtilis* PS-216 *wt* cells were cultured in liquid LB medium at 37°C and 200 rpm overnight. Overnight culture was diluted 100 times in 20 mL of fresh LB medium and incubated further at the same growth conditions. OD_650_ was measured in regular intervals during the culture incubation. At appropriate OD_650_ in different growth phases [i.e., middle exponential (OD_650_ ∼ 0.7), late exponential (OD_650_ ∼ 1.1) and stationary phase OD_650_ ∼ 1.3], the strains were treated with 5.9 mg L^-1^ of prodigiosin. As a control, an equivalent amount of sterile 96% (V/V) ethanol was added to the LB medium in the final concentration 0.19% (V/V).

### Treatment of Different Bacterial Strains with Prodigiosin

*Bacillus mycoides* DSM2048 cells were cultured in liquid LB medium at 28°C and 200 rpm overnight, while *B. licheniformis* ATCC9445A cells were cultured in liquid LB medium at 37°C and 200 rpm overnight. Overnight cultures were diluted 100 times in 20 mL of fresh LB medium and incubated further at the same growth conditions. Cultures were treated with prodigiosin (1.2 mg L^-1^ *B. mycoides* or 6.9 mg L^-1^ *B. licheniformis*) or ethanol as a control [0.04% (V/V) *B. mycoides* or 0.23 % (V/V) *B. licheniformis*] in the middle exponential phase at OD_650_ between 0.4 and 0.5. Treated cells were then incubated further and OD_650_ was measured in regular intervals during 6 h of incubation. To determine the influence of prodigiosin on different strains, the rate of lysis was calculated from measured decrease of OD_650_ after prodigiosin treatment.

### Cell Morphology and Viability

*Bacillus subtilis* PS-216 *wt* and *B. subtilis* PS-216 *amyE::*P_hyperspank_*-mKate2* strains were grown in LB medium as described above and treated with 5.9 mg L^-1^ of prodigiosin or 0.19% (V/V) of ethanol in early exponential phase at OD_650_ between 0.4 and 0.5. Cell morphology and viability were inspected by Differential Interference Contrast (DIC) and fluorescence microscopy using appropriate filter set under the inverted microscope Axio Observer Z1 (Carl Zeiss, Germany) at 0, 1, and 5 h after the prodigiosin or ethanol treatment.

### Effect of Prodigiosin on Inactive Cells

*Bacillus subtilis* PS-216 *wt* cells were grown in LB medium as described above. When cells reached OD_650_ between 0.4 and 0.5, bacterial culture was autoclaved at 121°C and 1.03 bar for 20 min to inactivate cells. After autoclaving, cell suspension was cooled down to room temperature and 5.9 mg L^-1^ of prodigiosin was added. Cell suspension was incubated further at 37°C and 200 rpm and OD_650_ was measured every 30 min for 5 h.

### Cytoplasmic Membrane Leakage

Cytoplasmic membrane leakage was determined as β-galactosidase activity according to the modified method by Mensa et al. (2011). Briefly, *B. subtilis* PS-216 *srfA-lacZ* (*neo)* cells were grown overnight in LB medium with appropriate antibiotic at 37°C and 200 rpm and diluted 100 times in 20 mL of the fresh CM medium. Cells were incubated at 37°C and 200 rpm until they reach OD_650_ ∼ 0.6. At that point 70 μL of cells were transferred in a microtiter plate and mixed with 20 μL of Z buffer (60 mM Na_2_HPO_4_, 40 mM NaH_2_PO_4_, 10 mM KCl, 10 mM MgSO_4_, pH 7) supplemented with 4 mg mL^-1^ ONPG (*o*-nitrophenyl-β-galactoside) and 40 μM β-mercaptoethanol. Cells were then treated with 10 μL of prodigiosin to a final concentration of 2.9 mg L^-1^. Microtiter plates were incubated in a spectrometer for 2 h at 37°C and absorbance at 420 nm was measured every 2 min to determine the rate of ONPG hydrolysis. The addition of 10 μL of 0.1% (w/V) sodium dodecyl sulfate (SDS) detergent [final concentration 0.01% (w/V)] represented a positive control, while the addition of 10 μL of 96% (V/V) ethanol [final concentration 9.6% (V/V)] represented a negative control. To calculate specific enzyme activity, the rate of ONPG hydrolysis was normalized to the cell biomass.

### Bacterial Response to Different Autolysin Inducing Agents

*Bacillus subtilis* PS-216 *wt* strain was grown in LB medium as described above and treated with 0.0125% (V/V) Triton X-100, 0.01% (w/V) SDS, 0.01% (w/V) cetyl trimethylammonium bromide (CTAB), 100 μg mL^-1^ ampicillin or 100 μg mL^-1^ kanamycin in the middle exponential phase at OD_650_ between 0.4 and 0.5. Ampicillin was used as a positive control known to induce cell lysis, whereas kanamycin was used as a negative control (Chung et al., 2009; Falk et al., 2010; Lacriola et al., 2013). Cell suspension was incubated further at 37°C and 200 rpm and OD_650_ was measured every 30 min for 5 h. To determine the impact of different detergents on bacterial strain, the rate of lysis was calculated from measured decrease of OD_650_ after treatment with antimicrobial agents.

### Autolytic Response of Bacterial Strains to Prodigiosin

*Bacillus subtilis* strains PS-216 *wt*, PS-216 *ΔlytABC*, PS-216 *ΔlytABCD*, PS-216 *ΔlytABCDE* and PS-216 *ΔlytABCDEF* were grown in LB medium as described above and treated with 5.9 mg L^-1^ of prodigiosin or 0.19 % (V/V) of ethanol as a negative control at OD_650_ between 0.4 and 0.7. Cells were then incubated further and OD_650_ was measured in regular intervals during 6 h of incubation at 37°C and 200 rpm. To determine the influence of prodigiosin on different mutant strains, the rate of lysis was calculated from measured decrease of OD_650_ after prodigiosin treatment.

### Modified Comet Assay

*Bacillus subtilis* strains PS-216 *wt*, PS-216 *ΔlytABC*, PS-216 *ΔlytABCD*, PS-216 *ΔlytABCDE* and PS-216 *ΔlytABCDEF* were grown in LB medium as described above. At OD_650_ between 0.4 and 0.6 cells were treated with 5.9 mg L^-1^ of prodigiosin or 0.19% (V/V) of ethanol. Cells were then incubated further for 1 h at 37°C and 200 rpm to lyse. Minigels were prepared on frosted-end microscopic slides. Slides were first dipped into 1% (w/V) normal melting point agarose (NMP) and one side was wiped clean. After drying on a hot plate (1 min, 100°C), the second layer of 300 μL 1% (w/V) NMP agarose was applied and solidified at 4°C for 5 min. For the third layer treated or non-treated cells were resuspended in 1% (w/V) low melting agarose (ratio 1:10) and left to solidify for 5 min. Minigels were immediately electrophoresed in TBE (Tris/borate/EDTA buffer, pH 8.3) at 1 V/cm and run for 30 min. Afterward minigels were neutralized in 400 mM Tris buffer (pH 7.4). Gels were stained with 30x solution of GelRed^TM^ (Biotium, USA) and observed under epifluorescent microscope (Olympus BX-50, Japan) equipped with EMCCD camera (Luca^EM^r, Andor Technology Ltd., United Kingdom) and the appropriate filter set. Images were captured by Komet 7.0 software (Andor Technology Ltd., UK).

### Data Analysis

All the values presented are averages with standard deviations or standard errors. Results were statistically evaluated using one-way ANOVA. Samples with *p*-values equal or lower than 0.05 were taken as statistically significant.

## Results And Discussion

Prodigiosin isolated from *V. ruber* DSM14379 inhibits growth of different bacterial species. The MIC value for *Escherichia coli* is 103.4 ± 6.3 mg L^-1^, while different *Bacillus* sp. strains tested have significantly lower MIC values. The MIC values for different *Bacillus* sp. strains were in the range between 5 and 7 mg L^-1^ (MIC values for *B. amyloliquefaciens* FZB42 was 6.1 ± 1.2 mg L^-1^, *B. licheniformis* ATTC9445A was 6.9 ± 2.2 mg L^-1^, *B. mycoides* was 1.2 mg L^-1^, *B. subtilis* NCIB3610 was 5.2 ± 1.4 mg L^-1^, *B. subtilis* ATCC6051 was 5.9 mg L^-1^ and *B. subtilis* PS-216 was 5.9 mg L^-1^) and were lower as previously reported (Stankovic et al., 2012; Priya et al., 2013). Approximately the same MIC values for different *Bacillus* species may suggest that prodigiosin has the same mode of action on all tested *Bacillus sp.* Only undomesticated natural isolate *B. subtilis* PS-216 *wt* was used for further analysis with MIC value 5.9 mg L^-1^. To test the *in vivo* activity of prodigiosin on *B. subtilis* PS-216 *wt* simple co-cultures with prodigiosin producing *V. ruber* DSM14379 were made. There was a significant decrease in the number of *B. subtilis* cells after incubation in co-culture. The Malthusian fitness of *B. subtilis* grown in a monoculture was 5.7 ± 0.2, but was significantly lower -6.8 ± 1 in co-culture experiments. The concentration of prodigiosin in co-culture at the end of the experiment was 16.3 ± 0.4 mg L^-1^ which is 2.7 fold higher than the MIC required for growth inhibition of *B. subtilis* PS-216 *wt*. The results indicate that prodigiosin has *in vivo* antimicrobial activity.

**FIGURE 1:**
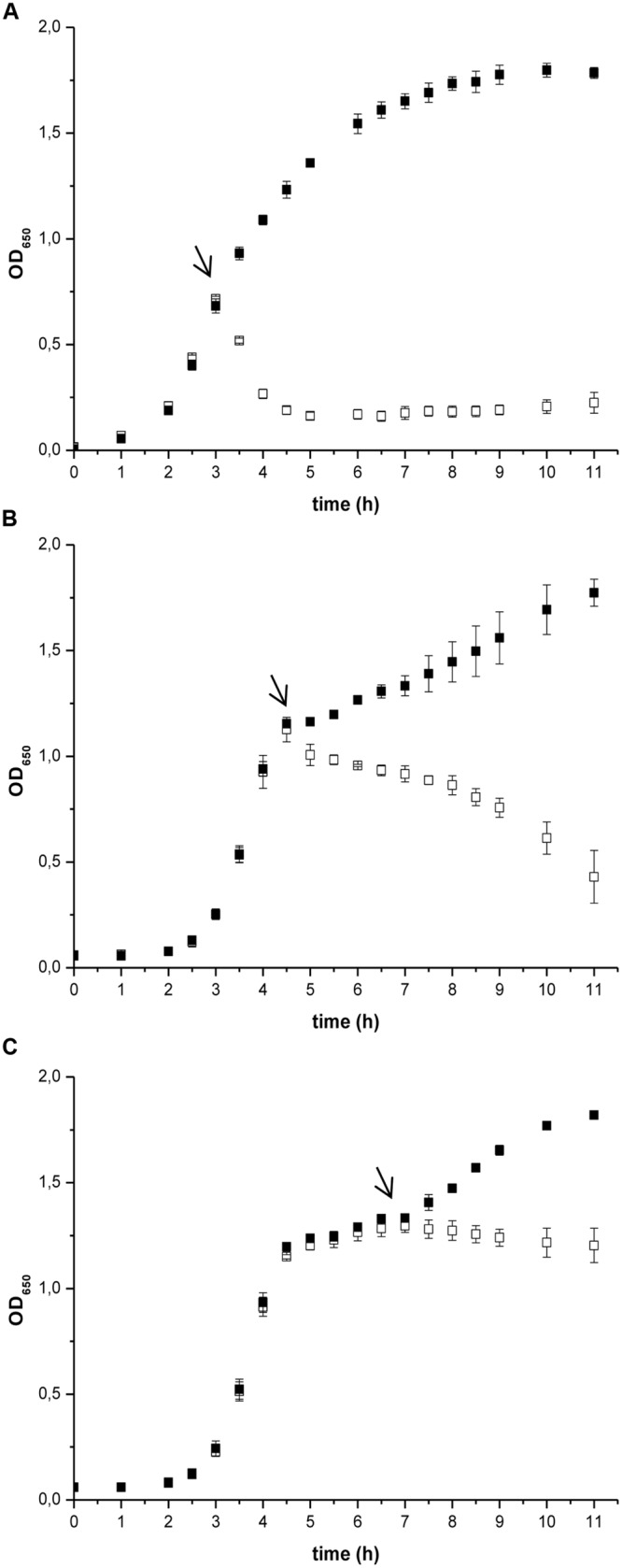
**The effect of prodigiosin addition on *Bacillus subtilis* PS-216 *wt* growth in LB medium at 37°C and 200 rpm at different growth phases.** The arrows represent the time of addition of 5.9 mg L^-1^ prodigiosin (opened squares) or 0.19% (V/V) ethanol in the control samples (filled squares). **(A)** cells treated in the middle of the exponential phase; **(B)** cells treated in the late exponential phase; **(C)** cells treated in the stationary phase. Data are presented as averages and standard deviations (*n* = 3).

**FIGURE 2:**
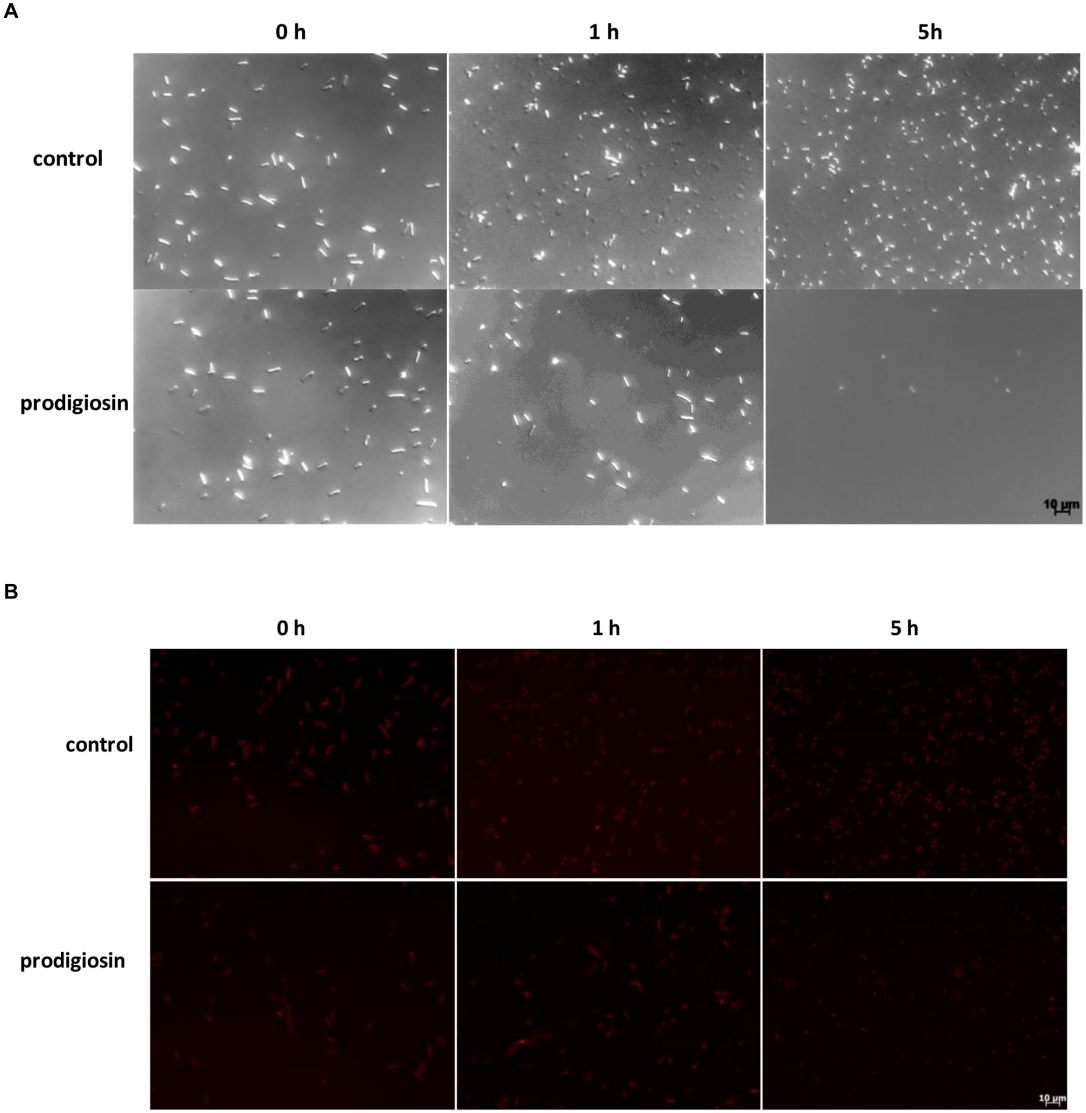
***Bacillus subtilis* PS-216 *wt* cells **(A)** and *B. subtilis* PS-216 *amyE*::P*_hyperspank_*-*mKate2***(B)** grown in LB medium at 37°C and 200 rpm observed by DIC and fluorescent microscopy at 0, 1, and 5 h after the addition of either 5.9 mg L^-1^ of prodigiosin or 0.19% (V/V) of ethanol in control samples in the middle of the exponential phase.** The scale bar represents 10 μm.

The effect of prodigiosin on *B. subtilis* was dependent on the growth phase as shown in **Figure 1**. Prodigiosin treatment in the middle of the exponential phase (**Figure 1A**) caused instantaneous termination of growth and immediate decrease of optical density. Cell lysis was completed in 2 h after prodigiosin addition. As shown in **Figure 2** cell number significantly decreased after prodigiosin treatment. After 5 h of incubation with prodigiosin the bacterial solution was clear and only a few bacteria were observed. Cells treated with prodigiosin in the late exponential phase (**Figure 1B**) lysed as well. However, the rate of lysis was three times lower than in the middle of the exponential phase. After 5 h of prodigiosin treatment, optical density decreased for approximately 50%. When cells were treated with prodigiosin in the stationary phase (**Figure 1C**) their growth ceased, but cells did not lyse. When those cells were re-inoculated into a fresh medium and incubated at optimal growth conditions, they resumed grow immediately. This implies that the mode of action of prodigiosin on *B. subtilis* cells depends on the growth phase. During the exponential growth prodigiosin has a bacteriolytic activity, whereas in the stationary phase it has bacteriostatic activity. This finding is not surprising, as it is generally accepted that effectiveness of antibiotic is greatly dependent on bacterial growth rate (Hobby et al., 1942; Greulich et al., 2015). For example, β-lactam antibiotics that target cell wall have their maximum effect during fast bacterial growth and are not effective toward stationary cells (Eng et al., 1991). The requirement of cell growth for prodigiosin susceptibilty was further tested with inactivated cells from the middle of the exponential phase. There was no significant change in optical density during 5 h of the incubation with prodigiosin, suggesting that the inactive cells did not lyse (data not shown). After the addition of the control containing ethanol (**Figure 1**), the growth of *B. subtilis* continued. Although the culture was not significantly diluted (the total volume increased only by 0.2%), the added ethanol may have had a positive effect on the growth of *B. subtilis* as demonstrated previously (Gomaa, 2014).

**FIGURE 3:**
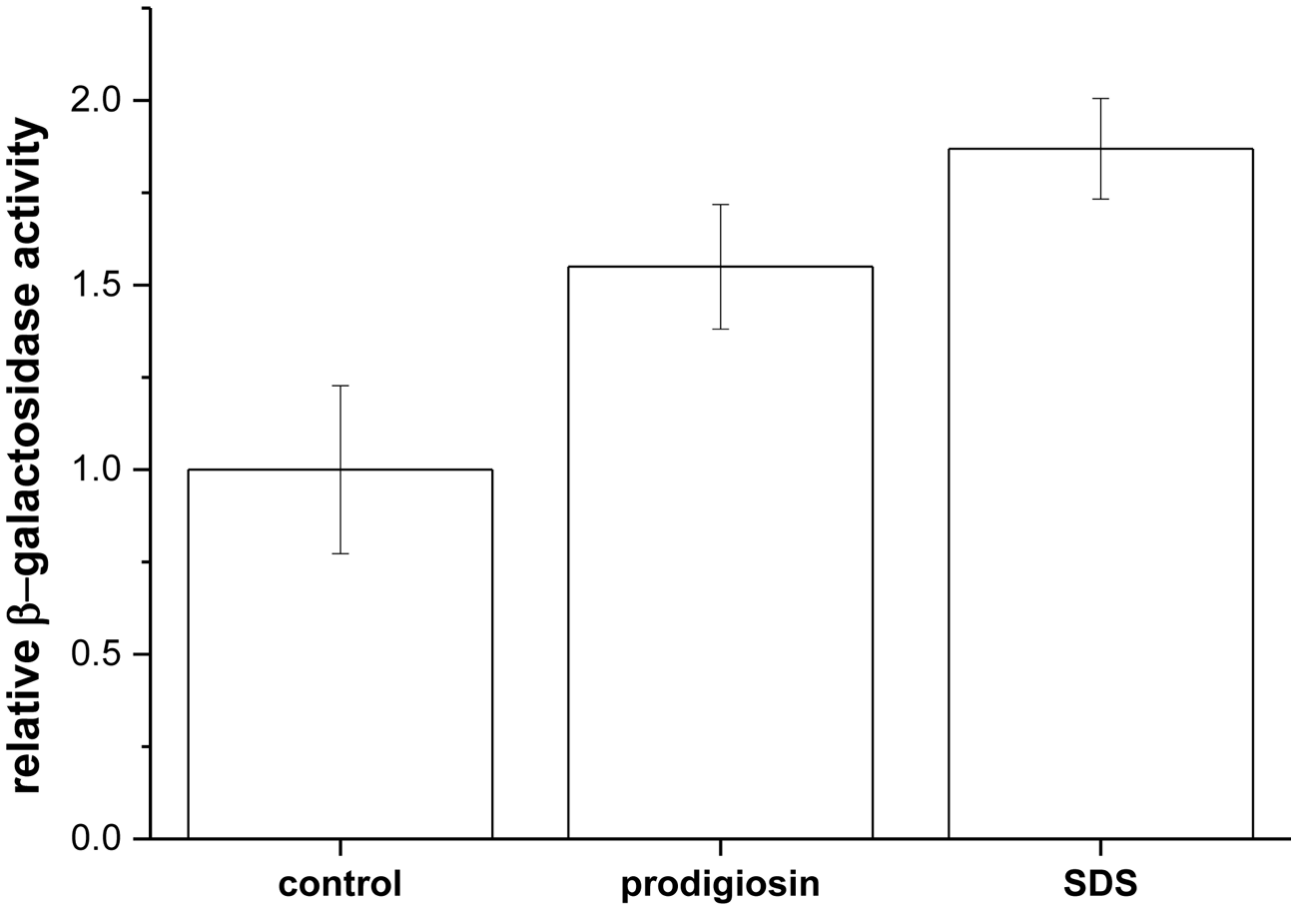
**Relative β-galactosidase activity upon addition of 5.9 mg L^-1^ prodigiosin and 0.01% (w/V) SDS in *B. subtilis* PS-216 *srfA-lacZ* (*neo*) cells compared with control [addition of 0.19% (V/V) ethanol].** Cells were grown in LB medium at 37°C and 200 rpm. Data are presented as averages and standard errors (*n* = 3–6).

For cell lysis one expects both cell wall and membrane disintegration. Prodigiosin is a small molecule that can diffuse through the cell wall and due to its amphiphilic character it can incorporate in the membrane (Diaz et al., 2007). Membrane loaded with prodigiosin may be compromised and allow leakage of cell constituents. To test cytoplasmic membrane permeability ONPG hydrolysis by β-galactosidase was used. ONPG can enter the cell and reach cytoplasmatic β-galactosidase only if cytoplasmic membrane is compromised. As given in **Figure 3** prodigiosin was able to permeabilize cytoplasmic membrane. Similar permeabilization was observed with SDS detergent, which is a known cytoplasmic membrane solubilization agent in *B. subtilis* and *Streptococcus faecalis* (Bishop et al., 1967; Cornett and Shockman, 1978). There were no statistically significant differences between prodigiosin and SDS treatment (*p* = 0.4). The results indicate that prodigiosin interacts with cytoplasmic membrane and increases its permeability. Increased cytoplasmic permeability was associated with autolysis in *B. subtilis* (Falk et al., 2007). It has been shown that proton-motive force influences regulation of autolysins in *B. subtilis* cells (Calamita and Doyle, 2002). Prodigiosin functions in an energy-spilling process as a tightly regulated uncoupler of proton transport and ATP synthesis by oxidative phosphorylation (Haddix et al., 2008).

When cells were treated with prodigiosin in the middle of the exponential phase, approximately half of the cells lysed in 45 min. This lytic rate of prodigiosin (0.37 ± 0.01 h^-1^) is comparable to cell lysis induced by Triton X-100 (0.36 ± 0.03 h^-1^) and ampicillin (0.49 ± 0.06 h^-1^), which is a known autolysin induction agent (Lacriola et al., 2013). On the other hand, the other known agents that induce autolysins (i.e., SDS and CTAB) had lower lytic rates. SDS had lytic rate 0.13 ± 0.01 h^-1^, while CTAB had lytic rate 0.15 ± 0.01 h^-1^. Kanamycin known as a non-autolysin agent did not lyse *B. subtilis*, the lytic rate was – 0.12 ± 0.03 h^-1^. The results indicate that there was a significant difference between lytic rate of prodigiosin and SDS, which is different from the β-galactosidase results (**Figure 3**). Whereas β-galactosidase release assay shows the cytopasmic membrane permeabilisation, the lytic rate shows autolysin induction. The two are not necessarily coupled. For example, SDS is a typical solubilizing agent of cytoplasmic membrane proteins in *B. subtilis* (Bishop et al., 1967), but is usually not used as an autolysin inducer due to its low lytic effectiveness. It has been demonstrated in *Enterobacteriacea* that the SDS-grown cells underwent rapid lysis only when they ran out of energy (Kramer et al., 1980). The energy dependence reflects a requirement for ATP rather than for a proton gradient or membrane potential (Aspedon and Nickerson, 1994). Prodigiosin, on the other hand, acts as a potent lytic agent for *B. subtilis* that uncouples proton transport in the cytoplasmic membrane (Haddix et al., 2008), thereby activating autolysins. Prodigiosin lytic activity was not limited to *B. subtilis* cells only. For example, treating *B. mycoides* and *B. licheniformis* with prodigiosin at MIC concentrations induces cell lysis and the cell suspension cleared after 20 h of treatment. The corresponding lytic rates were 0.014 ± 0.002 h^-1^ for *B. mycoides* and 0.009 ± 0.001 h^-1^ for *B. licheniformis*, which indicates that prodigiosin lytic activity could be a more general mechanism in *Bacillus* species.

**FIGURE 4:**
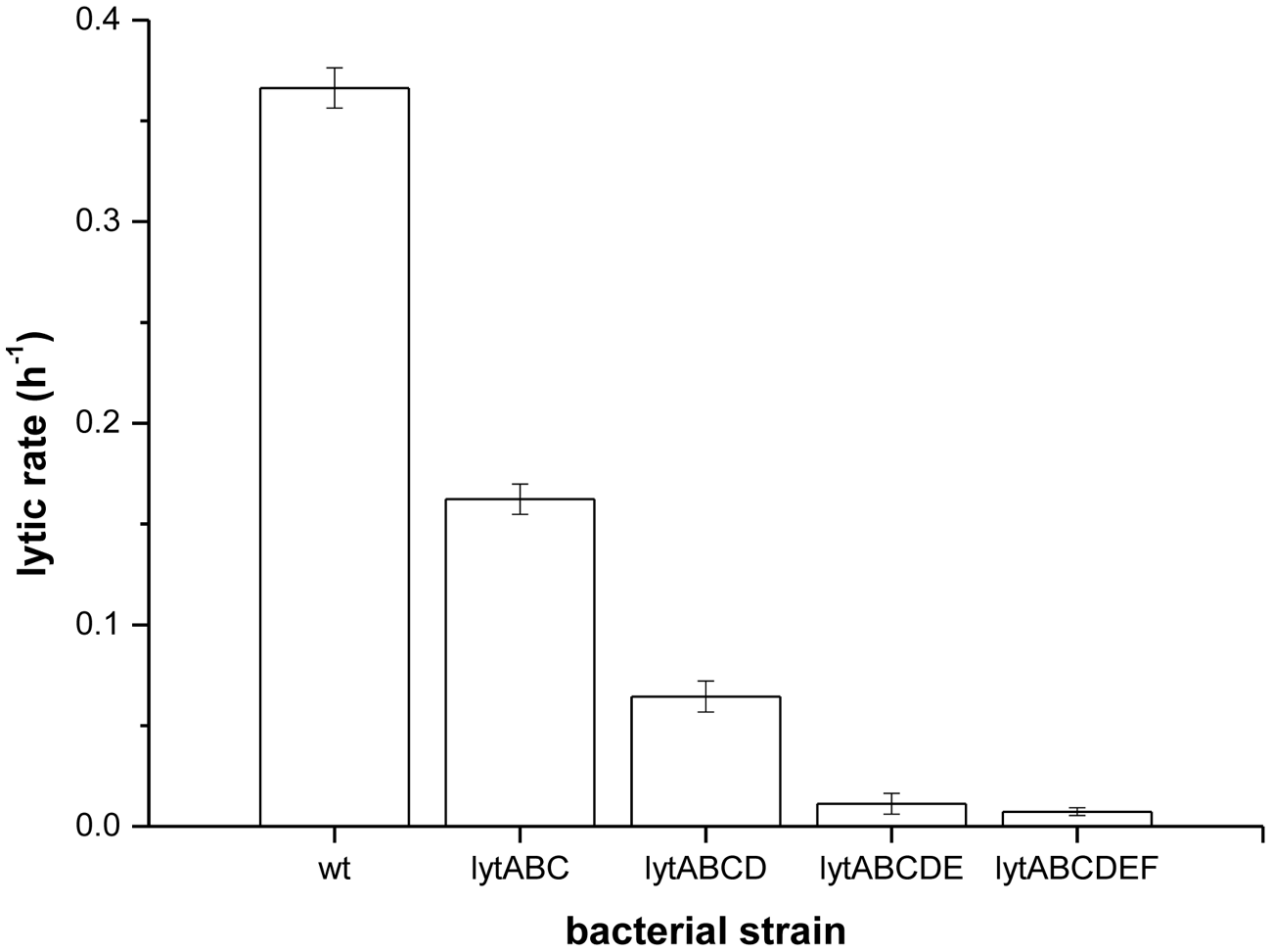
**Lytic rates of different *lyt* mutants measured as a rate of OD_650_ decrease after treatment with prodigiosin (5.9 mg L^-1^) in LB medium at 37°C and 200 rpm.** Data are presented as averages and standard deviations (*n* = 3).

To prove that prodigiosin induces autolysis several mutant strains of *B. subtilis* PS-216 deficient in major autolysis genes (*lyt* genes) were produced. The lytic rates after treatment with prodigiosin of mutant strains deficient to a different extent in autolytic response are given in **Figure 4**. In the *lytABC* deficient strain the cell lysis was significantly reduced and the lytic rate was approximately two fold lower compared to the wild type strain. When additionally *lytD* gen was deleted a further decrease in the lytic rate was observed. The *lytABCD* deficient strain showed approximately 80% reduction of lysis compared to the wild type strain. This is consistent with previous reports of a key role of LytC and LytD in autolysis (Smith et al., 2000). In addition, it has been shown previously that *lytE* mutant may significantly reduce autolysis (Margot et al., 1998). Consistently in *lytABCDE* mutant there was almost no lysis after prodigiosin addition. Finally, in *lytABCDEF* mutant the lytic rate upon addition of prodigiosin was 50 times lower compared to the wild type strain. Although no lytic response was observed in *lytABCDEF* mutant the addition of prodigiosin in the middle of exponential phase had a bacteriostatic effect. The results obtained by different *lyt* deficient strains strongly suggest that prodigiosin induces autolysin dependent bacteriolysis in *B. subtilis*. For a complete lysis of *B. subtilis* by prodigiosin a complement of different autolysins is needed. Since autolysins can be induced also by other amphiphatic molecules such as short- and medium–chain fatty acids (Tsuchido et al., 1985) this further supports the hypothesis that prodigiosin is a potent autolysin modulator.

**FIGURE 5:**
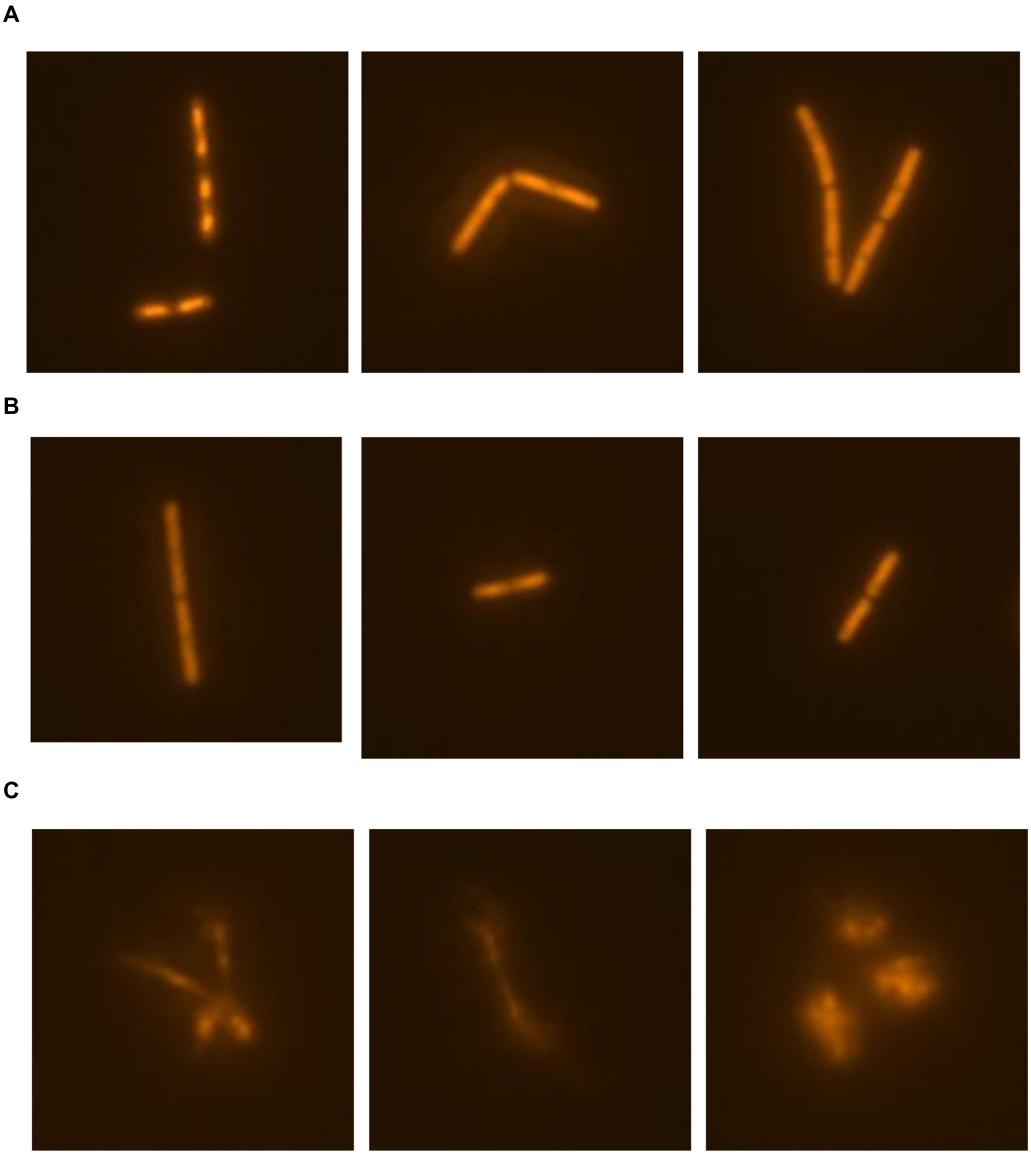
**The modified comet assay for studying prodigiosin mediated cell lysis of *B. subtilis* PS-216 *wt* cells grown in LB medium at 37°C and 200 rpm. (A)** non-treated bacterial cells; **(B)** control cells treated with 0.19% (V/V) of ethanol; **(C)** cells treated with 5.9 mg L^-1^ of prodigiosin. Cells were treated in the middle exponential phase and were inspected 1 h after the treatment. The gels were stained with GelRed^TM^. The images were observed with epifluorescent microscopy.

**FIGURE 6:**
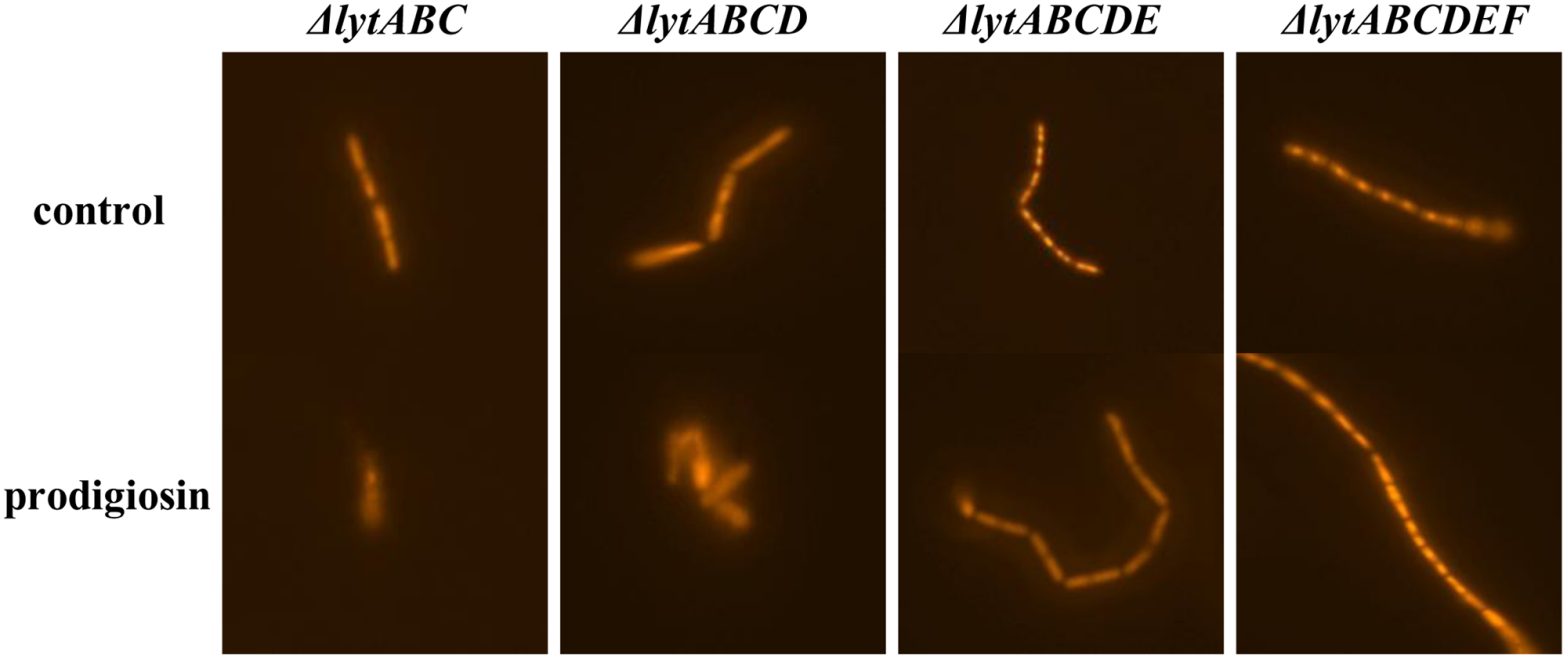
**The modified comet assay on different autolysin deficient strains of *B. subtilis* PS-216 cells grown in LB medium at 37°C and 200 rpm treated with 0.19% (V/V) of ethanol (control) or treated with 5.9 mg L^-1^ of prodigiosin.** Cells were treated in the middle exponential phase and were inspected 1 h after the treatment. The gels were stained with GelRed^TM^. The images were observed with epifluorescent microscopy.

The release of nucleic material during cell lysis was followed with modified comet assay (**Figure 5**). Non-treated cells retained cell integrity through the experiment, displaying clearly defined shape without tail or halo (**Figure 5A**). Similary, in control cultures treated with 0.19% (V/V) ethanol, the same concentration as used for prodigiosin solubilization, cells were intact (**Figure 5B**). On the other hand, treatment with prodigiosin induced instant lysis in almost all examined cells (**Figure 5C**). Cells that lysed appeared blurred with rapid decline of fluorescence indicating DNA diffusing out of disrupted cells. The comet assay was also applied to *B. subtilis* mutant strains deficient in major autolysin genes. *Lyt* mutants treated with prodigiosin responded differently. The comet assay suggests that lysis was most intense in *lytABC* mutant. The degree of cell lysis decreased from *lytABC* to *lytABCDEF* deficient strain (**Figure 6**). Negligible cell lysis was observed in *lytABCDEF* mutant strain after prodigiosin treatment. The results obtained with the modified comet assay confirm that prodigiosin induces rapid autolysis in *B. subtilis* cells.

Finally, we note that upon entry into the stationary phase prodigiosin acts as a bacteriostatic agent. During transition to the stationary phase *B. subtilis* cells begin to lose their ability to move and start to produce biofilm. Biofilms and motility are mutually exclusive lifestyles of *B. subtilis* (Diethmaier et al., 2011) and are controlled by a common regulator, the transcription factor SinR. During biofilm formation SinR interacts with SinI and de-represses synthesis of extracellular matrix important in biofilm formation. One of the components of extracellular matrix EpsE interacts with flagellar motor switch protein FliG and prevents rotation of the flagellum (Blair et al., 2008). In addition, upon the entry into a stationary phase cells shut down synthesis of flagella components. The synthesis of flagella and autolysin are under the same regulation control. The synthesis of flagella proteins requires genes which are under the control of alternative RNA polymerase sigma factor, σ^D^ (Shahbabian et al., 2009). σ^D^ is a pleiotrophic transcription regulator during the exponential growth and has a positive effect on autolysin synthesis (Fein, 1979). Upon entry into the stationary phase σ^H^ decreases the expression of σ^D^ (Britton et al., 2002), which in turn down-regulates autolysin production. In the absence of autolysins, as demonstrated in this work, the lytic activity of prodigiosin is abolished. However, as shown with *lytABCDEF* mutant prodigiosin may still act as a bacteriostatic agent preventing further growth of *B. subtilis*.

## Conclusion

The application of prodigiosin as an antibacterial agent is hampered by the lack of knowledge of its molecular targets. The results of this study demonstrate that prodigiosin exhibits a potent antimicrobial activity against *B. subtilis*. It acts as a bacteriolytic agent during the exponential growth and as a bacteriostatic agent during the stationary growth. Prodigiosin interferes with cytoplasmic membrane function and increases its permeability. The obtained results indicate that prodigiosin’s bacteriolytic activity is due to the induction of autolysins. Prodigiosin proves to be a strong autolysin inducer in *B. subtilis* cells comparable in its activity to Triton X-100. Since prodigiosin functions as an uncoupler of proton transport and ATP synthesis, it is ideally suited for autolysins induction and killing of different *Bacillus* species.

## Author Contributions

Conceived and designed the experiments: TD, MV, and DS. Performed the experiments: TD, MV, MT, and MZ. Analyzed the data: TD, MV, MT, and MZ. Wrote the paper: TD, MV and DS.

## Conflict of Interest Statement

The authors declare that the research was conducted in the absence of any commercial or financial relationships that could be construed as a potential conflict of interest.
