# Supplementary material for: Prodigiosin Induces Autolysins in Actively Grown Bacillus subtilis Cells
Source: Front Microbiol. 2016 Jan 28;7:27. doi: 10.3389/fmicb.2016.00027 (PMC4729933; doi:10.3389/fmicb.2016.00027)
Supplement: Supplementary file 1 [file Presentation_1.PDF]

Supplementary Material

PRODIGIOSIN INDUCES AUTOLYSINS IN ACTIVELY GROWN *Bacillus subtilis* CELLS

Tjaša Danevčič[1], Maja Borić Vezjak[1], Maja Tabor[1], Maša Zorec[2], David Stopar[1,*]

*** Correspondence:** David Stopar: david.stopar@bf.uni-lj.si

Supplementary Figure

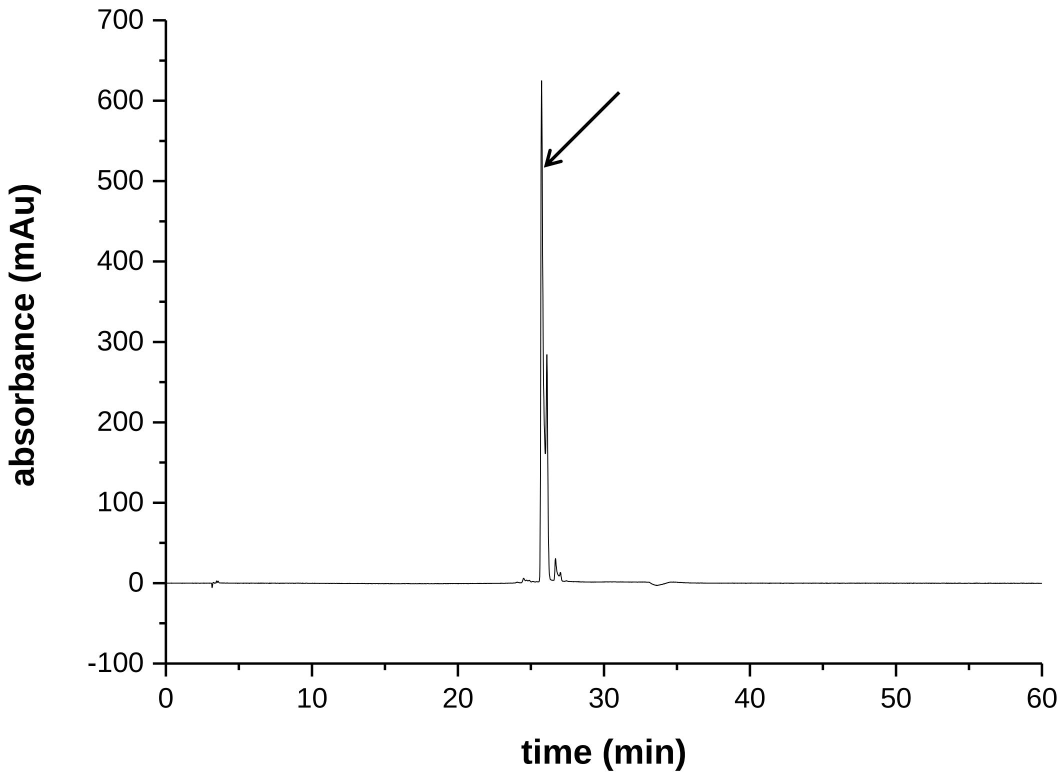

Supplementary Figure 1. HPLC elugram of prodigiosin extract from *Vibrio ruber* DSM14379. Absorbance was measured at 535 nm, The prodigiosin peaks were marked with black arrow. The purity of the prodigiosin is 98 % as determined by HPLC.
